# Pyridoxine-Responsive Seizures in Infantile Hypophosphatasia and a Novel Homozygous Mutation in ALPL Gene

**DOI:** 10.4274/jcrpe.2798

**Published:** 2016-09-01

**Authors:** Banu Güzel Nur, Gamze Çelmeli, Esra Manguoğlu, Erdoğan Soyucen, İffet Bircan, Ercan Mıhçı

**Affiliations:** 1 Akdeniz University Faculty of Medicine, Department of Pediatric Genetics, Antalya, Turkey; 2 Akdeniz University Faculty of Medicine, Department of Pediatric Endocrinology, Antalya, Turkey; 3 Akdeniz University Faculty of Medicine, Department of Medical Biology, Antalya, Turkey; 4 Akdeniz University Faculty of Medicine, Department of Pediatric Metabolism, Antalya, Turkey

**Keywords:** Infantile hypophosphatasia, ALPL gene, Novel mutation, pyridoxine responsive

## Abstract

Hypophosphatasia is a rare inherited disorder of bone and mineral metabolism caused by a number of loss-of-function mutations in the ALPL gene. It is characterized by defective bone and tooth mineralisation associated with low serum and bone alkaline phosphatase activity. The clinical presentation of this disease is extremely variable. For this reason, the diagnosis can be difficult and is often missed out or delayed. Hypophosphatasia is classified into subtypes based on the age of onset and clinical features. The clinical severity is associated with the age at diagnosis and the lack of tissue-nonspecific alkaline phosphatase activity; the severe forms of hypophosphatasia are primarily perinatal and infantile forms. Severe forms may present with many neurological problems such as seizures, hypotonia, irritability. Herein, we report the case of an infantile hypophosphatasia patient who presented with pyridoxine-responsive seizures and a novel homozygous mutation in the ALPL gene was detected. There is a limited number of hypophosphatasia patients with pyridoxine-responsive seizures in the literature, so early diagnosis of infantile hypophosphatasia in the clinically compatible patients allows more effective postnatal care/management and genetic counseling for further pregnancies.

**WHAT IS ALREADY KNOWN ON THIS TOPIC?**

There is a limited number of hypophosphatasia patients with pyridoxine-responsive seizures in the literature.

**WHAT THIS STUDY ADDS?**

We reported the case of an infantile hypophosphatasia patient who presented with pyridoxine-responsive seizures. A novel homozygous mutation in the ALPL gene was detected.

## INTRODUCTION

Hypophosphatasia (HPP) [Online Mendelian Inheritance in Man (OMIM) 146300, 241500 and 241510] is a hereditary congenital bone disease marked by a deficiency of alkaline phosphatase (ALP) activity in the liver, bones, and kidneys and is associated with defective skeletal mineralization. It was first described in 1948 by Rathbun (1). HPP has diverse phenotypes and is classified into seven subtypes based on the age of onset and clinical features: perinatal, benign prenatal, infantile, childhood and adult type, odontohypophosphatasia, and pseudohypophosphatasia (2). There are several patterns of inheritance. As already stated, inheritance can be autosomal dominant or recessive, but de novo mutations have also been described (3). Severe forms of HPP, primarily perinatal and infantile, are inherited in an autosomal recessive manner. Moderate forms (mHPP), primarily prenatal benign, childhood, adult, and odontohypophosphatasia, mostly result from heterozygosity for dominant severe alleles or from compound heterozygosity for severe and moderate alleles, and more rarely from two moderate alleles. The prevalence of severe HPP was estimated at 1/300,000, and prevalence of dominant mHPP was estimated to be 1/6370 in the European population (4).

HPP is caused by loss-of-function mutations in the ALPL gene, the gene encoding the isoenzyme tissue-nonspecific ALP (TNSALP), which is located in the chromosome region 1p36.1-1p34. It consists of 12 exons distributed over 50 kb. Many mutations have been described on the TNSALP gene, mostly in European, North American, and Japanese patients (5).

The clinical severity is associated with the age at diagnosis and the lack of TNSALP activity, except for odontohypophosphatasia where only the teeth are affected. Patients with the perinatal form of HPP almost always die around birth due to impaired development of the lungs and the severe hypomineralization of their bones (6). Infantile type symptoms are similar to, but typically less severe than, perinatal form and are recognized before 6 months of age. Childhood- and adult-onset HPP typically present with premature loss of deciduous teeth, rachitic changes in children and osteopenia, recurrent fractures, and pseudofractures with early loss of adult dentition, common osteomalacia in adult, and delayed healing of fractures (7). In this manuscript, we present an infantile patient diagnosed with HPP who had a novel homozygous mutation in the ALPL gene.

## CASE REPORT

**Figure 1:**
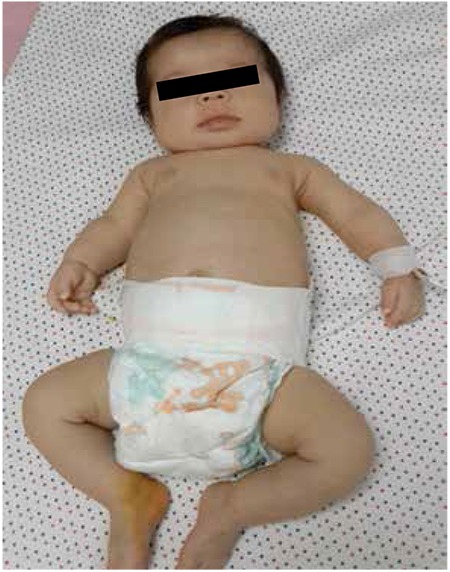
Facial phenotype of the patient showing flattened facial appearance, broad forehead, flattened nasal bridge, bilateral low-set ears, short neck, and narrow thorax

A one-month-old female infant was evaluated because of limb shortening, hypercalcemia, and epilepsy. She was born at the 39th week of gestation by Caesarean section with a birthweight of 2070 g as the first child of consanguineous parents. Her prenatal ultrasound and family medical history were normal. The APGAR score was 7 at 1 minute and 10 at 5 minutes after birth. Metabolic acidosis and seizures were observed in the first day of life. Intubation was attempted because of respiratory failure. She was referred to our hospital because of myoclonic seizures that were not controlled after phenobarbital and phenytoin therapy. On her physical examination at one month old, her weight was 2470 g (<3^th^ centile), her height was 50.5 cm (3^th^-10^th^ centile), and her head circumference was 35.5 cm (10^th^-50^th^ centile). A flattened facial appearance, broad forehead, flattened nasal bridge, bilateral low-set ears, short neck, narrow thorax, shortening of left arm and dimples on knees, hepatomegaly palpable 2 cm below the right costal margin, and 2/6 systolic murmur were detected (Figure 1).

**Figure 2:**
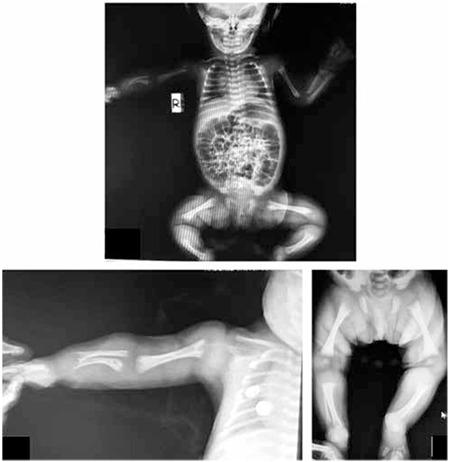
Radiographic imaging of the patient showing distorted trabeculation, reduced mineralization, metaphyseal irregularities, cupping, diaphyseal shortening, shortness of the right humerus, ulna, and radius

Her skull and long bones radiographies showed distorted trabeculation, reduced mineralization, metaphyseal irregularities, cupping, diaphyseal shortening, shortness of the right humerus, ulna, and radius (Figure 2). Craniosynostosis was not observed. Her echocardiography detected ostium secundum atrial septal defect. Renal ultrasonography revealed medullary hyperechogenicity suggesting medullary nephrocalcinosis. Her ophthalmologic examination, cranial magnetic resonance imaging, and electroencephalography were normal.

The results of blood serum tests were as follows: calcium 14.5 mg/dL (N: 8.8-10.2), phosphorus 7.1 mg/dL (N: 4.5-5.5), ALP 1 IU/L (N<70), magnesium 1.8 mg/dL (N: 1.7-2.55), parathyroid hormone (PTH) 5.5 pg/mL (N: 15-65), and 25-hydroxy vitamin D_3_ 49.5 ng/mL (the cut-off for vitamin D3 deficiency is <20). Urinary calcium excretion was high with a calcium/creatinine ratio of 0.64. Her complete blood count and routine biochemistry tests for renal, liver, thyroid functions, immunoglobulins, tandem mass spectrometry, urinary and plasma amino acid analysis, urinary organic acid analysis were all found to be within normal limits.

Her seizures, refractory to previous antiepileptic therapy, were under control after pyridoxine (vitamin B6) administration (100 mg/day initial, 50 mg/day maintenance dose, PO). Intravenous hydration with saline plus furosemide was started at the time of admission to treat hypercalcemia, and dietary calcium intake was restricted. This therapy had minimal effect on calcium levels, and serum calcium was 13.8 mg/dL on the 3rd hospital day. The hypercalcemia was resistant to other treatment options including prednisolone (2 mg/kg/d). Normocalcemia was achieved with a single dose of calcitonin (4 U/kg/d).

**Figure 3:**
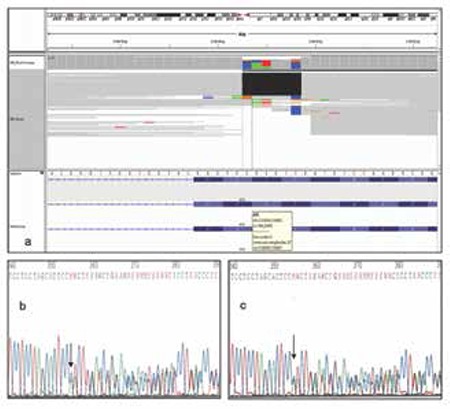
Diagram showing homozygous deletion (c.799_840delCACTTC) on ALPL gene of the proband (a). Electropherograms of father (b) and mother (c) confirming heterozygous presence of the same mutation that was detected in the proband

Chromosome analysis of peripheral leukocytes using high-resolution binding technique showed a normal 46,XX karyotype. A homozygous p.267_268delHF (c.799_804delCACTTC) mutation was detected in ALPL gene. The parents were also heterozygous for the new mutation. Genomic DNA of both parents and the index patient was screened for mutations in ALPL gene, and Sanger-sequencing revealed a novel homozygous mutation, p.267_268delHF (c.799_804delCACTTC), in the index patient (Figures 3a, 3b, 3c). This alteration was not annotated in National Center for Biotechnology Information or the human gene mutation database, and both parents were heterozygous carriers for this novel mutation in ALPL gene.

## DISCUSSION

The clinical presentation of HPP is variable and is thought to reflect the severity of the mutation in the ALPL gene as well as the mode of inheritance (dominant vs. recessive). The clinical characteristics of infantile type of HPP are respiratory complications, premature craniosynostosis, demineralization, rachitic changes in the metaphyses, hypercalcemia, short stature, and premature loss of primary teeth (8). Clinical features, age, bone mineralization, elevated serum concentrations of calcium and phosphorus, and low serum ALP enzyme activity helps differentiate HPP from other conditions. Differential diagnosis of infantile HPP includes osteogenesis imperfecta type 2, thanatophoric dysplasia, campomelic dysplasia, chondrodysplasia with bone mineralization defects. In addition, irritability, poor feeding, failure to thrive, hypotonia, and seizures place the infantile type in a broad differential diagnosis that includes inborn errors of energy metabolism, organic acidemia, and non-accidental trauma.

HPP is a distinct variant of rickets or osteomalacia and due to the block of mineral uptake into the skeleton, hypercalcemia and hyperphosphatemia are common in infantile HPP. Low serum PTH levels, normal serum 25-hydroxy vitamin D and 1,25-dihydroxy vitamin D, and hypercalciuria are the other typical biochemical findings of the disease (9). Radiological findings may be similar to rickets. It can be distinguished from rickets by low ALP levels (10). In the presented patient, HPP was considered with onset before 6 months of age, phenotypic appearance, skeletal deformities, hypercalcemia, remarkably low level of ALP, and low PTH level. The phenotypes, extremities, as well as calcium and ALP levels were normal in both parents.

We also observed seizures refractory to antiepileptic therapy but responsive to pyridoxine. Only a few HPP patients were reported in the literature with pyridoxine-responsive seizures, and it was considered to be an indicator of disease severity. Several specific mutations have been suggested to be responsible. The mechanism of pyridoxine-responsive seizures in HPP is explained by defective metabolism of pyridoxal 5-phosphate (PLP), which is the phosphorylated form of pyridoxine. PLP, one of the natural substrates of ALP, is the active compound by which pyridoxine mediates essential enzyme activity; PLP deficiency in the central nervous system may reduce seizure threshold by reducing neurotransmitter (GABA) synthesis (11,12,13,14,15,16).

Molecular analysis of the ALPL gene can verify the genetic defect. A homozygous p.267_268delHF (c.799_804delCACTTC) mutation was detected in ALPL gene. To date, at least 210 distinct mutations and 12 polymorphisms have been reported. Most of the reported mutations (79.3%) are missense mutations, and the remaining reported mutations are deletions (small deletions 10.1%, large deletions 0.9%), splicing mutations (4.1%), nonsense mutations (2.8%), small insertions (1.8%), a complex deletion + insertion, a de novo mutation, and a nucleotide substitution affecting the major transcription initiation site (17). After searching the single nucleotide polymorphism database and the human gene mutation database, we found that p.267_268delHF (c.799_804delCACTTC) mutation is absent from the two databases; thus, our patient carry a novel missense mutation in the ALPL gene.

Although enzyme replacement therapy may provide a therapeutic option, still there is no current therapy for HPP. Recently, clinical trials have been undertaken using recombinant human TNSALP especially in a small number of infants and young children with severe HPP as well as in juveniles and adults, with promising results for bones, and pulmonary and physical functions (18). However, further investigation is needed in phenotype-genotype correlation studies to predict the severity of the disease, and clinical trials to evaluate treatment strategies for HPP. In our patient, when the health status stabilized, she was referred to another university hospital by air ambulance to receive enzyme replacement therapy.

In conclusion, infantile HPP has a high mortality rate, and it is important to consider the infantile HPP in the differential diagnosis of skeletal deformities, hypercalcemia, and low level of ALP. Molecular diagnosis is necessary to better understand the molecular basis of the disease, to improve the outcomes of genetic counseling, and may offer the possibility of future prenatal diagnosis.

## Ethics

Informed Consent: Informed consents were obtained from the patient parents.

Peer-review: Externally peer-reviewed.
